# A concept analysis of nurses in conflicts after World War II

**DOI:** 10.1111/jan.15454

**Published:** 2022-10-11

**Authors:** Anne M. Fink, Gwyneth R. Milbrath

**Affiliations:** ^1^ Department of Biobehavioral Nursing Science College of Nursing, University of Illinois Chicago Chicago Illinois USA; ^2^ Midwest Nursing History Research Center and Department of Population Health Nursing Science College of Nursing, University of Illinois Chicago Chicago Illinois USA

**Keywords:** concept analysis, deployment, emergency nursing, military, nurses' roles, peace, post‐traumatic stress, war

## Abstract

**Aim:**

We analysed nurses' experiences during military conflicts since World War II.

**Background:**

Nurses have successfully reduced morbidity and mortality in populations affected by wars; despite centuries of nurses' global involvement in wars, there is limited knowledge about their experiences.

**Method:**

We used Rodger's evolutionary concept analysis methodology to understand the antecedents, attributes, consequences, context and implications of nurses' war‐related experiences. We analysed data from quantitative and qualitative research, media reports, editorials, historical reviews and published accounts of nurses' experiences in many locations, including Afghanistan, Bosnia, Croatia, Korea, Kosovo, Iran, Iraq, Israel, Palestine, Russia, Somalia, Ukraine and Vietnam.

**Findings:**

Two antecedent conditions preceded nurses' war involvement: *actively responding to human suffering* and *having resources for readiness*. Nurses were defined by five attributes: *sacrifice*, *resourcefulness*, *tunnel‐vision*, *survival mindset* and *comradery*. We also found evidence for seven consequences; nurses saved lives (*reduced morbidity and mortality*), however, some nurses faced *professional burnout/disillusionment*, *restricted practice authority*, *isolation* and *post‐traumatic stress* after war. In addition, *growth* and *pacifism* were consequences for some nurses who were exposed to war.

**Conclusion:**

The findings of our concept analysis illustrate how nurses have fulfilled critical life‐saving roles, but some nurses' post‐war experiences were debilitating, stigmatized and unsupported. We conclude that research about the resourcefulness, innovations and resiliency nurses have developed during wars is essential, and professional support mechanisms must be developed to prevent post‐traumatic stress, burnout and attrition from the profession. Governments can use utilize the knowledge nurses developed during wars to expand emergency preparedness skillsets and promote nurses as the leaders of international efforts to promote peace.

**No Patient or Public Contribution:**

Patients, service users, caregivers and members of the public were not involved in conducting this concept analysis or preparing the manuscript.

**Impact statement:**

- By understanding nurses' involvement with post‐WWII conflicts, we have demonstrated the significant public health contributions, challenges and personal and professional growth experienced by nurses.
- Nurses' war‐related knowledge should be utilized to innovate healthcare practices during disasters and to advise policymakers in developing, implementing and evaluating peace‐promoting operations.

## 1 INTRODUCTION

Wars—armed conflicts among governments or paramilitary groups—cause tremendous suffering, death and destruction. War was defined by Griffiths and Jasper (2008) as the “antithesis of health,” physically, psychologically and economically. Nurses have cared for war‐afflicted populations by providing emergency care, supporting humanitarian missions and implementing measures to prevent infectious and food/water‐borne illnesses (Keeling & Wall, 2015). The boundaries of nursing knowledge, practice and authority have been pushed forward by the challenges that are inherent in wars and disasters (Milbrath, 2019). Wars have also shaped the nursing profession's norms, achievements, and histories, over centuries, and around the world (Milbrath, 2019). Despite nurses' impactful efforts in supporting the health of civilians and combatants, most research endeavours about wars have overlooked nurses' experiences.

World War II (WWII) represents a pivotal event in the history of nursing. The utilization of antibiotics, surgical asepsis and blood banking profoundly advanced nursing practice during this era (Milbrath, 2019). Nursing degree programmes were established in universities around the world after WWII (Hazrati et al., 2011). Nurses' professional opportunities in the armed forces also expanded after WWII, particularly in the United States (U.S.) and United Kingdom (U.K.). Notably, a more diverse workforce was employed, and nurses could join the armed forces and receive military ranks, benefits, promotions and training (Milbrath, 2019). Before WWII, for example, American nurses were contractors employed by the military to care for wounded and ill soldiers. When the U.S. Army and Navy Nurse Corps were established at the turn of the 20th century, the Nurse Corps remained siloed from the rest of the medical department, which withheld military training from nurses. The nurse was only provided with relative military ranks and limited opportunities for advancement. Significantly, only white women were permitted to join the Nurse Corps before and during WWII—men and women of colour were barred from serving, with rare exceptions (Milbrath, 2019). Considering how the nursing workforce changed, and nurses became more autonomous and educated after WWII, the purpose of the present paper was to analyse nurses' war‐related experiences only during the post‐WWII era.

## 2 METHODS

### 2.1 Design and data collection

**FIGURE 1:**
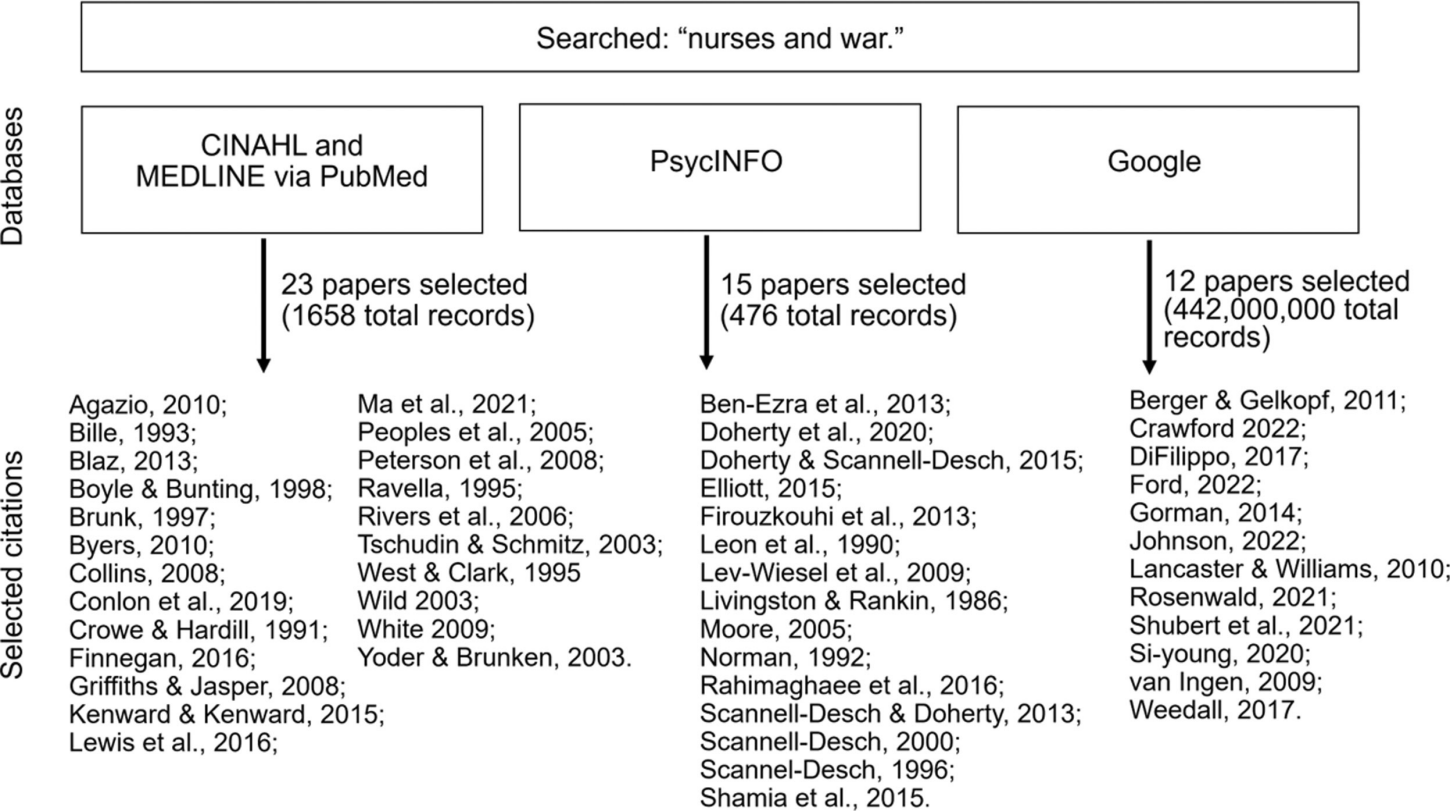
Literature search strategy.

Concept analyses, according to Rodgers (2000), are inductive knowledge‐building exercises that must be performed before researchers can develop hypotheses, measures or interventions for future studies. Rodger's concept analysis methodology provides a systematic approach for defining a concept and its contextual factors. Rodgers encouraged investigators to analyse data from many different types of source material (e.g., research, news, literature or art), and she recommended obtaining at least 30 records to conduct a valid assessment. Consistent with these instructions, we designed a search strategy that would provide us with a combination of scientific literature, personal accounts and media reports. We conducted the literature search in April 2022. We used a purposive sampling method, similar to Ames et al. (2019), to collect a variety of articles about nurses' war‐related experiences that would reflect data from different perspectives, locations, nationalities and post‐WWII time periods. As shown in Figure 1, we used the terms “nurses and war” to search multiple databases: the Cumulative Index to Nursing and Allied Health Literature (CINAHL) and MEDLINE (via PubMed) and PsycINFO. We also entered “nurses and war” into Google where we selected eight news articles, two letters, one newsletter and one research paper. Using these databases, we selected 50 articles that reflected nurses' experiences with wars and conflicts, globally. To be included in the analysis, papers were required to report the experiences of nurses who were deployed or living in areas impacted by war. We included papers about Military Operations Other Than War (MOOTW), defined as humanitarian missions and military activities designed to deter impending wars, as long as the nurses were engaged with frequent mass casualty events in regions experiencing sustained violence (e.g., Operation Restore Hope in Somalia). We excluded depictions of nurses from fictional literature or from sources designed for entertainment (e.g., M*A*S*H, *China Beach*) because fictional characters may not reflect nurses' factual experiences and characteristics. In the present paper, we use the terms “war” and “conflict” interchangeably.

### 2.2 Data analysis

To analyse the articles, we read each paper twice while compiling a list of the common themes. After this reading phase, the themes were categorized as antecedents (pre‐existing conditions), attributes (clusters of conceptual definitions), or consequences (outcomes), which collectively described nurses' experiences with war (Table 2). We adhered to Rodgers' (2000) definition of concepts—she argued that concepts “are formed by the identification of characteristics common to a phenomenon” (p. 78).

Rodgers also emphasized the importance of considering a concept's “contextual basis.” The contextual basis refers to the “temporal” and “sociocultural variations” of concepts (p. 85). Temporal variations consider the impact of time (referring to the conceptual antecedents and consequences) but also illustrate Rodgers' view that conceptual definitions often evolve over time (p. 85). Sociocultural variations explain how cultures, religions and nationalities may influence the definitions of a concept in different situations. To address these aspects of Rodgers' methodology, we reported the characteristics of nurses in each study (Table 1), such as their locations, nationalities, religions, genders, employment and education (when these data were available); this information was evaluated to determine whether the times, locations or nurses' characteristics altered any conceptual attributes.

**TABLE 1:** Summary of articles used for the concept analysis

Sources of data (citations)	Location(s) of wars/conflicts	Description (research designs; types of papers)	Sample sizes (number of nurses or perspectives)	Nurse characteristics and perspectives			
				Military or civilian	Genders	Religions	Professionalization (ranks/training/education)
Agazio (2010)	Afghanistan, Bosnia, Columbia, Guatemala, Haiti, Honduras, Hungary, Iraq, Kosovo, Kuwait, Philippines, Peru, Saudi Arabia Virgin Islands	Research (qualitative; interviews) about clinical challenges	75	Military	Women (58%) and Men (42%)	Not reported	U.S. Army Officers (92% active duty; 8% reservists). 57% had bachelor's degrees, 42% had master's degrees and 1% had doctorates
Ben‐Ezra et al. (2013)	Israel	Research (quantitative; survey of randomly sampled hospital nurses) about post‐war symptoms	46	Civilian	Women (91%) and Men (9%)	Not reported	Not reported
Berger and Gelkopf (2011)	Israel	Research (quantitative; quasi‐randomized controlled trial) testing a 12‐wk educational intervention to prevent burnout	90	Civilian	Women (100%)	Secular Jewish (45%), Traditional Jewish (31%), Religious Jewish (15%), Muslim (9%)	All subjects were well‐baby clinic nurses (education data were not reported)
Bille (1993)	Vietnam	Author's perspectives about post‐traumatic stress disorder	1	Military	Man	Not reported	U.S. Army Officers
Blaz et al. (2013)	Afghanistan, Iraq	A review about training	*N/A*	Military	*N/A*	*N/A*	U.S. Military Officers
Boyle and Bunting (1998)	Iran, Iraq	An editorial opposing war	*N/A*	Civilian	*N/A*	*N/A*	*N/A*
Brunk (1997)	Iraq, Vietnam	Review paper about the history of wartime nursing	*N/A*	Military	*N/A*	*N/A*	*N/A*
Byers (2010)	Afghanistan, Iraq	Review about clinical practice (e.g., transfusion, resuscitation and tourniquet application)	*N/A*	Military	*N/A*	*N/A*	U.K. Military Officers
Collins (2008)	Iraq, Kosovo	Review paper about critical care transport teams	*N/A*	Military	*N/A*	*N/A*	U.S. Military Officers
Conlon et al. (2019)	Not specified	Research (qualitative; hermeneutic phenomenological methodology) about lived experiences of officers	6	Military	Women and men (% not reported)	Not reported	Australian Army and Navy Officers
Crawford (2022)	Ukraine	News story about volunteers in Ukraine	*N/A*	Civilian	*N/A*	Not reported	Volunteers
Crowe and Hardill (1991)	Iraq	Editorial opposing war	*N/A*	Civilian	*N/A*	*N/A*	Canadian Nurses
DiFilippo (2018)	Vietnam	News story/interviews	3	Military	Women (100%)	Not reported	U.S. Military Officers
Doherty et al. (2020)	Afghanistan, Iraq	Research (mixed methods) about post‐war experiences	278	Military	Women (76%) and men (24%)	Not reported	Officers in U.S. Air Force, Army, and Navy nurses on active duty, reservists, or in the National Guard
Doherty and Scannell‐Desch (2015)	Afghanistan, Iraq	Research (qualitative; phenomenological methodology) about reintegration post‐deployment	35	Military	Women (91%) and men (9%)	Not reported	Officers in U.S. Air Force, Army, and Navy nurses on active duty, reservists, or in the National Guard
Elliott (2015)	Afghanistan, Iraq	Research (qualitative; narrative inquiry) about post‐deployment experiences	10	Military	Women (70%) and men (30%)	Not reported	U.S. Army (80%) and Air Force (20%) Officers
Finnegan et al. (2016)	Afghanistan	Research (qualitative; constructivist grounded theory) about values/characteristics	18	Military	Women (44%) and men (56%)	Not reported	U.K. Armed Forces Nurses (72% Officers [28% other ranks]); 72% Army, 22% Royal Air Force, 6% Royal Navy
Ford (2022)	Ukraine	Anti‐war letter from nurses	Unknown	Civilian	Not reported	Not reported	Not reported
Firouzkouhi et al. (2013)	Iran	Research (historical) about Iran‐Iraq War nursing	15	Civilian	Men (100%)	Not reported	92% were experienced nurses (77% had previously been members of the armed forces before becoming nurses); 13% had an undergraduate degree in nursing
Gorman (2004)	Afghanistan, Iraq	Editorial opposing war	3	Civilian	Women (100%)	Not reported	American Nurses
Griffiths and Jasper (2008)	Bosnia, Iraq	Research (qualitative; grounded theory design) about nurses' experiences of war	24	Military	Not reported	Not reported	U.K. Royal Navy, Army and Royal Air Force Officers
Johnson (2022)	Ukraine	News story/interview	1	Civilian	Woman	Not reported	Ukrainian Nurse Practitioner
Kenward and Kenward (2015)	Afghanistan, Iraq	Literature review about nurses' deployment experiences	*N/A*	Military	Not reported	Not reported	North American and U.K. Military Nurses
Lancaster and Williams (2010)	Afghanistan	Newsletter describing Forward Surgical Teams	*N/A*	Military	*N/A*	*N/A*	U.S. Army Officer
Leon et al. (1990)	Vietnam	Research (mixed method) about post‐war experiences	36	Military	Women (100%)	Not reported	Not reported
Lev‐Wiesel et al. (2009)	Israel	Research (quantitative/questionnaires) about post‐traumatic stress	76	Civilian	Women (88%) and men (12%)	Jewish (89%), Druze or Muslim (8%), Christian (4%)	Not reported
Lewis et al. (2016)	Afghanistan, Iraq	Review paper about nurse practitioners	*N/A*	Military	*N/A*	Not reported	U.S. Army Officers
Livingston and Rankin (1986)	Korea, Vietnam	A case study and feminist analysis of war	1	Military	*N/A*	*N/A*	American Nurses
Ma et al. (2021)	Afghanistan, Bosnia, Iran, Iraq, Kosovo, Vietnam	Review and qualitative meta‐analysis about experiences before, during, and after deployments	*N/A*	Military	Not reported	Not reported	Military Nurses from Multiple Countries (e.g., Australia, Iran, Korea, Sweden, U.K. and U.S.)
Moore (2005)	Not specified	Commentary about psychiatric advanced practice nursing	*N/A*	Military	*N/A*	*N/A*	Advanced Practice Nurses
Norman (1992)	Vietnam	Research (qualitative) about nurses' identities and careers	50	Military	Women (100%)	Not reported	American Vietnam War Veterans (education data were not reported; some nurses pursued advanced degrees after deployment)
Peoples et al. (2005)	Afghanistan	Paper surgical teams, causalities, and outcomes	*N/A*	Military	*N/A*	*N/A*	U.S. Army Officers (Forward Surgical Team)
Peterson et al. (2008)	Iraq	Paper about psychiatric nursing	*N/A*	Military	*N/A*	*N/A*	U.S. Military and Advanced Practice Psychiatric Nurses
Rahimaghaee et al. (2016)	Iran, Iraq	Research (qualitative) about experiences during war	14	Not specified	Women (79%) and men (21%)	Not reported	Iranian nurses with ≥3 months exposure to war (education data were not reported)
Ravella (1995)	Vietnam	Research (mixed methods) about war experiences	20	Military	Women (90%) and men (10%)	Not reported	American Vietnam War Veterans
Rivers et al. (2006)	N/A	Research (quantitative) about training	131	Military	Women (44%) and men (56%)	Not reported	U.S. Military Officers (60%) and Enlisted Personnel (40%; levels of education and experienced varied)
Rosenwald (2021)	Vietnam	News story/interview	1	Military	Woman	Not reported	U.S. Army Officer
Scannell‐Desch and Doherty (2013)	Afghanistan, Iraq	Research (qualitative) about deployment and parenting	20	Military	Women (80%) and men (20%)	Not reported	U.S. Army, Navy and Air Force Officers
Scannell‐Desch (2000)	Vietnam	Research (qualitative) about hardships and strategies	24	Military	Women (100%)	Not reported	U.S. Army Vietnam War Veterans
Scannell‐Desch (1996)	Vietnam	Research (qualitative) about post‐deployment experience	24	Military	Women (100%)	Not reported	U.S. Army Vietnam War Veterans
Shamia et al. (2015)	Palestine	Research (quantitative) about post‐traumatic stress	274	Civilian and Military	Women (47%) and men (53%)	Not reported	Nurses were working for the Ministry of Health (79%), the United Nations Relief and Works Agency (10%), Military Medical Services (5%) and the private sector (6%).
Shubert (2021)	Afghanistan	News story/interview	*N/A*	Military	Woman	*N/A*	U.S. Army Officer
Si‐young (2020)	Korea	News story/interview	1	Military	Woman	*N/A*	Swedish Red Cross Officer
Tschudin and Schmitz (2003)	Afghanistan, Kosovo, Iraq, Vietnam	Anti‐war paper	*N/A*	Civilian	*N/A*	*N/A*	U.K. Nurse Authors
van Ingen (2009)	Korea	News story/interview	1	Military	Woman	Not reported	U.S. Army Korean War Veteran
Weedall (2017)	Korea	News story/interview	1	Military	*N/A*	Not reported	U.S. Army Korean War Veterans
West and Clark (1995)	Somalia	Paper summarizing interviews	*N/A*	Military	*N/A*	Not reported	U.S. Army Officers and Historian
Wild (2003)	Iraq and Vietnam	Literature review paper about post‐war experiences	*N/A*	Military	*N/A*	Not reported	Not reported
White (2009)	Iraq	Personal account about practice challenges	1	Military	Man	Not reported	U.S. Army Officer
Yoder and Brunken (2003)	Kosovo, Somalia	Review about nurses' roles in supporting peace‐keeping and humanitarian missions	N/A	Military	*N/A*	*N/A*	U.S. Army Officers

**TABLE 2:** Concept analysis results by article

Citations	Antecedents		Attributes					Consequences						
	Respond to human suffering	Resources for readiness	Sacrifice	Resource‐fulness	Tunnel‐vision	Survival mindset	Comradery	Reduced morbidity/mortality	Professional burnout/disillusionment	Restricted practice authority	Isolation	Post‐traumatic stress	Growth	Pacificism
Agazio (2010)	**X**		**X**	**X**		**X**								
Ben‐Ezra et al. (2013)												**X**		
Berger and Gelkopf (2011)									**X**					
Bille (1993)							**X**		**X**		**X**	**X**		
Blaz et al. (2013)				**X**				**X**						
Boyle and Bunting (1998)														**X**
Brunk (1997)	**X**											**X**		
Byers (2010)								**X**						
Collins, 2008		**X**						**X**						
Conlon et al. (2019)				**X**		**X**	**X**				**X**			
Crawford (2022)	**X**													
Crowe and Hardill (1991)														**X**
DiFilippo (2018)	**X**						**X**		**X**	**X**	**X**	**X**		
Doherty et al. (2020)												**X**	**X**	
Doherty and Scannell‐Desch (2015)									**X**		**X**	**X**		
Elliott (2015)									**X**		**X**	**X**	**X**	
Finnegan et al. (2016)			**X**		**X**	**X**	**X**						**X**	
Ford (2022)														**X**
Firouzkouhi et al. (2013)			**X**			**X**				**X**				
Gorman (2004)														**X**
Griffiths and Jasper (2008)		**X**		**X**	**X**	**X**								
Johnson (2022)	**X**													
Kenward and Kenward (2015)	**X**		**X**	**X**	**X**		**X**							
Lancaster and Williams (2010)				**X**				**X**						
Leon et al. (1990)							**X**					**X**		
Lev‐Wiesel et al. (2009)													**X**	
Lewis et al. (2016)		**X**						**X**						
Livingston and Rankin (1986)											**X**	**X**		**X**
Ma et al. (2021)					**X**		**X**				**X**	**X**		
Moore (2005)								**X**						
Norman (1992)									**X**	**X**				
Peoples et al. (2005)								**X**						
Peterson et al. (2008)								**X**						
Rahimaghaee et al. (2016)	**X**		**X**	**X**	**X**		**X**					**X**		
Ravella (1995)						**X**	**X**	**X**				**X**		
Rivers et al. (2006)						**X**								
Rosenwald (2021)					**X**									
Scannell‐Desch and Doherty (2013)			**X**	**X**	**X**						**X**			
Scannell‐Desch (2000)							**X**					**X**		
Scannell‐Desch (1996)	**X**		**X**	**X**	**X**		**X**			**X**	**X**	**X**	**X**	**X**
Shamia et al. (2015)												**X**		
Shubert (2021)					**X**									
Si‐young (2020)	**X**			**X**										
Tschudin and Schmitz (2003)														**X**
van Ingen (2009)							**X**							
Weedall (2017)			**X**											
West and Clark (1995)			**X**	**X**		**X**	**X**							
Wild (2003)	**X**								**X**		**X**	**X**		
White (2009)			**X**		**X**		**X**	**X**						
Yoder and Brunken (2003)			**X**	**X**			**X**					**X**		

## 3 RESULTS

Our analysis included information about conflicts in the following locations and times: Korea (1950–1953), Vietnam (1955–1975), Somalia (1992–1993), the Balkan Peninsula (conflicts in Bosnia, Croatia and Kosovo; 1992–1999), the Middle East (conflicts in Afghanistan, Iran, Iraq, Israel, Kuwait and Palestine; since 1990) and Ukraine (2022). Our sources included journalists' reports/interviews (Crawford, 2022; DiFilippo, 2017; Johnson, 2022; Rosenwald, 2021; Si‐young, 2020; van Ingen, 2009; Weedall, 2017), nurses' personal perspectives (Bille, 1993; Collins, 2008; White, 2009), newsletters (Lancaster & Williams, 2010), commentaries (Moore, 2005), and review papers (Blaz et al., 2013; Ma et al., 2021; Wild, 2003; Yoder & Brunken, 2003). Most of the papers presented the results of original research studies (Agazio, 2010; Ben‐Ezra et al., 2013; Berger & Gelkopf, 2011; Conlon et al., 2019; Doherty et al., 2020; Doherty & Scannell‐Desch, 2015; Elliott, 2015; Firouzkouhi et al., 2013; Griffiths & Jasper, 2008; Lev‐Wiesel et al., 2009; Rahimaghaee et al., 2016; Ravella, 1995; Scannell‐Desch, 1996; Scannell‐Desch, 2000; Scannell‐Desch & Doherty, 2013; Shamia et al., 2015). Several papers focused on the training for military nurses (Agazio, 2010; Blaz et al., 2013; Byers, 2010; Lewis et al., 2016; Peterson et al., 2008; Rivers et al., 2006; Yoder & Brunken, 2003). Not all of the nurses who authored papers were affiliated with militaries or chose to support war, which gave our analysis a range of perspectives—several nurses published anti‐war arguments (Boyle & Bunting, 1998; Crowe & Hardill, 1991; Ford, 2022; Gorman, 2004; Livingston & Rankin, 1986; Tschudin & Schmitz, 2003).

### 3.1 Antecedents

#### 3.1.1 Actively responding to human suffering

Unlike natural disasters, humans create and perpetuate wars, causing profound human suffering. Wars forced people into austere environments with limited access to clean water, food, electricity, medicine, safety and shelter. Nurses responded by caring for civilians and combatants who suffered from traumatic injuries, illnesses, dehydration, starvation, psychological distress and the chronic effects of extreme poverty (Agazio, 2010; Boyle & Bunting, 1998; DiFilippo, 2017; Kenward & Kenward, 2015; West & Clark, 1995; Yoder & Brunken, 2003). In some cases, nurses volunteered to travel to countries experiencing invasions and war, such as Ukraine, Vietnam and Korea (Johnson, 2022; Scannell‐Desch, 1996; Si‐young, 2020). Nurses deployed from Europe (Finnegan et al., 2016; Griffiths & Jasper, 2008; Kenward & Kenward, 2015; Ma et al., 2021; Si‐young, 2020; Wild, 2003), Australia (Conlon et al., 2019) and North America (Table 1), and nurses also described the suffering caused by attacks on their own hometowns in Iran (Firouzkouhi et al., 2013; Ma et al., 2021; Rahimaghaee et al., 2016), Israel (Ben‐Ezra et al., 2013; Berger & Gelkopf, 2011; Lev‐Wiesel et al., 2009), and Palestine (Shamia et al., 2015).

An Iranian nurse said “I was always present on the front, and voluntarily so, because I felt the urge to help. The only reason for my presence there and for tolerating the extreme war conditions was love… there were people who didn't have to be there, who could have simply stayed in their own cities, but they were there serving anyways” (Rahimaghaee et al., 2016). Another nurse who joined the U.S. military described being inspired by a compassionate surgeon's character on the popular American television show M*A*S*H, which led him to value “talented healthcare providers” who could “save” people (Collins, 2008).

#### 3.1.2 Resources/Readiness

There were no reported cases of nurses working alone; they were always a part of teams supported by existing institutions (e.g., hospitals and military units). Nurses who were educated, trained and licensed to work in nursing were available and ready to support healthcare/humanitarian missions. Nurses who were military officers often had advanced practice degrees (Table 1), and they could lead interdisciplinary teams, practice autonomously and accept a high degree of responsibility (Blaz et al., 2013; Collins, 2008; Griffiths & Jasper, 2008; Lewis et al., 2016).

### 3.2 Attributes

#### 3.2.1 Sacrifice

Nurses made sacrifices in their roles to protect patients. They expected themselves to be prepared for “self‐sacrifice and selfless service” (Yoder & Brunken, 2003), and they described being proud of their commitment and patriotism (Agazio, 2010; Firouzkouhi et al., 2013; Kenward & Kenward, 2015; Scannell‐Desch, 1996; West & Clark, 1995). An Iranian nurse explained “under the influence of the soldiers' self‐sacrifice, I was also sacrificing myself. This spirit seemed to dominate everyone everywhere at that time” (Rahimaghaee et al., 2016).

Nurses faced uncertainty and danger as they worked long, unpredictable schedules. One described ‘living in a twilight zone” because they were “permanently on‐call” (Kenward & Kenward, 2015). When patients required urgent surgeries and transfusions, nurses who had the same blood types quickly donated their blood (Agazio, 2010; Kenward & Kenward, 2015; Weedall, 2017). White (2009) described how nurses used their bodies to shield patients when mortars exploded in the emergency department of Ibn Sina Hospital in Baghdad. They continued working while facing risks within medical facilities, such as frequent bombings (Crawford, 2022;Kenward & Kenward, 2015; Rahimaghaee et al., 2016) or when they were attacked by patients (Agazio, 2010). A nurse in Iran said “we were giving our own lives for the job. Amid the fire of the mortars, we carried the wounded to be treated; you could say the atmosphere itself was encouraging us” (Rahimaghaee et al., 2016). You might be “making the ultimate sacrifice, your life,” explained an American nurse who had worked in Vietnam (Scannell‐Desch, 1996). American and U.K. nurses also described sacrificing time with their families when they deployed because they left their spouses and young children at home (Finnegan et al., 2016; Scannell‐Desch & Doherty, 2013).

#### 3.2.2 Resourcefulness

Supplies were limited, and nurses relied on their abilities to devise creative approaches to fulfil their caregiving roles (Conlon et al., 2019; Lancaster & Williams, 2010; Scannell‐Desch, 1996; West & Clark, 1995). The “harsh conditions encouraged innovations” (Rahimaghaee et al., 2016). Nurses described “improvising” (Kenward & Kenward, 2015; Si‐young, 2020), having to “figure out how to fix stuff” (Agazio, 2010), being “extremely adaptable” to “think out of the box” (Yoder & Brunken, 2003), and becoming “multi‐skilled” (Griffiths & Jasper, 2008) and “self‐reliant” (Kenward & Kenward, 2015). An American nurse explained that “if you can fabricate things to make your job easier that's probably a good skill set to have,” and wartime experiences were described as “an incredible learning experience” (Agazio, 2010).

#### 3.2.3 Tunnel‐vision

Nurses worked with intense and focused attention; they followed their instincts, ignored the surrounding dangers and eliminated unnecessary distractions (Kenward & Kenward, 2015; Ma et al., 2021; Scannell‐Desch, 1996). They acknowledged experiencing anxiety and sadness but diverted attention towards their immediate tasks. White (2009) explained how nurses began working under flashlights in Baghdad when generators failed, and Shubert (2021) described a flight nurse delivering a baby while evacuating civilians from Kabul. Tunnel‐vision allowed nurses to make decisions rapidly and to function under suboptimal conditions, but it did not hinder their abilities to be compassionate and empathetic (Finnegan et al., 2016; Griffiths & Jasper, 2008; White, 2009). A *Washington Post* article quoted a former U.S. Army nurse saying “you just wanted to cry, but we also had a job to do” when describing her role in Operation Baby Lift, a mission that evacuated children from Vietnam in 1975 (Rosenwald, 2021). Another American nurse who served in Vietnam explained that she “never thought about the danger until it was all over.” She described the evacuation planes returning with “holes in them; we would land, and you could see some of the fire in certain areas, but you just didn't dwell on it” (Scannell‐Desch, 1996).

Nurses who cared for enemy combatants described their ability to ignore their underlying cultural and political differences, which prevented distractions from providing high‐quality nursing care; they made considerable efforts to reassure and communicate with patients when there were language barriers (Kenward & Kenward, 2015; Rahimaghaee et al., 2016). A few accounts described how caring for injured children could be the most difficult task because it could disrupt nurses' focus, especially when they lacked experience in paediatric nursing. In these situations, nurses asked colleagues who had paediatric nursing experience to care for children while they focused on adults—avoiding paediatric cases was a conscious decision to circumvent the vicarious trauma nurses expected to experience if a child died under their care (Finnegan et al., 2016; Scannell‐Desch & Doherty, 2013). In a study of American military nurse officers, some nurses described seeing their “own [children] in every little injured kid.” A nurse described how the “wounded children wouldn't hit [her] right away” but would trigger nightmares about the harms that could befall her own child; these nurses “swapped assignments” so they could maintain the *tunnel‐vision* their roles required (Scannell‐Desch & Doherty, 2013).

#### 3.2.4 Survival mindset

Nurses learned how to survive (Agazio, 2010; Ravella, 1995; West & Clark, 1995). Agazio (2010) explained that nurses developed an attitude for survival (defined as a mentality that protected themselves and their patients), and the survival mindset allowed nurses to “make the best of” their roles during bad situations. Deployments involved prolonged work hours, sometimes more than 30 h of continuous duty (Agazio, 2010; West & Clark, 1995). The work could require up to 15 continuous days without a break (Firouzkouhi et al., 2013). At times, mass casualty‐level traumas alternated with long periods of boredom—a survival mindset was required to endure the long, unpredictable and tumultuous schedules.

Nurses in the British Royal Navy, Army and Royal Air Force described how they had the training and psychological abilities to switch between their roles as nurses and their responsibilities as military officers. In the later roles, they had to make decisions about surviving potential dangers encountered at roadblocks and be prepared to fire towards the enemy. To engage in these dual roles (nurse and military officer), they compartmentalized their work into their functions for caregiving versus tasks necessary for survival (Griffiths & Jasper, 2008). An Australian nurse officer explained that “when the adrenaline kicks in, your military training just kicks in” (Conlon et al., 2019).

Even though nurses addressed the importance of survival, research conducted by Rivers et al. (2006) indicated that many American nurses required more pre‐deployment training in survival skills. Their research showed that nurses had high abilities for skills like map/compass reading and land navigation but lower skills for using military communications equipment and evading enemy capture (Rivers et al., 2006). A study of British military nurses revealed that they needed more pre‐deployment training about the local cultures so they could be better equipped in their interactions with civilians and prisoners of war (Finnegan et al., 2016). Information from American nurses of the 42nd and 46th Combat Support Hospitals indicated that they also required guidance about complicated survival‐related ethical issues that arose during Operation Restore Hope in Somalia. For example, there was a limited supply of ventilators, and the nurses feared having to make impossible decisions if numerous patients required respiratory life support (West & Clark, 1995).

#### 3.2.5 Comradery

Nurses relied on each other. They focused on their roles within teams instead of their individual selves (Conlon et al., 2019; Kenward & Kenward, 2015; Ma et al., 2021; Rahimaghaee et al., 2016; Scannell‐Desch, 1996; Yoder & Brunken, 2003). Experienced nurses viewed themselves as mentors who had responsibilities to fulfil in supporting novice nurses (Conlon et al., 2019; Ravella, 1995). An American nurse interviewed by Yoder and Brunken (2003) explained that “you are only somebody because of the person next to you. A chain is only as strong as its weakest link.” Nurses from the U.K. explained that their self‐worth was “aligned to strong team integration” while they were working in Afghanistan (Finnegan et al., 2016). Socializing was important—American nurses described “bonding” in Vietnam and having a “closeness different than any other relationship” (Scannell‐Desch, 1996). A British nurse explained that “from a team dynamics perspective it is important to see each other and to be part of each other's lives” (Finnegan et al., 2016).

Maintaining a shared sense of “humour” was a key component of nurses' comradery (Leon et al., 1990; Ravella, 1995; Scannell‐Desch, 1996). Joking and laughing with colleagues allowed nurses to collectively cope with fear, anger and distressing events in Vietnam, Somalia and Iraq (DiFilippo, 2017; Scannell‐Desch, 1996; Scannell‐Desch, 2000; West & Clark, 1995; White, 2009). For American nurses in Korea and Vietnam, “partying” provided a respite from work and an opportunity to build comradery; several accounts described how alcohol was often available in Korea and Vietnam (Bille, 1993; Scannell‐Desch, 2000; van Ingen, 2009). Developing close social connections came with psychological risks, however, as illustrated by the recollections of nurses whose colleagues were injured or killed (Bille, 1993; Conlon et al., 2019). An American nurse who worked in a Mobile Army Surgical Hospital in Korea explained how morale was also affected by the leadership qualities of their commanding officers—she explained an instance where poor leadership caused low morale, which she suspected led to two suicides during her deployment (van Ingen, 2009).

### 3.3 Consequences

#### 3.3.1 Reduced Morbidity and Mortality

The literature illustrated how nurses saved many lives during wars. According to White (2009), the survival rate for patients admitted to a U.S. Army Combat Support Hospital and Air Force Theatre Hospital was approximately 97% in Iraq. American nurses staffed Forward Surgical Teams and Critical Care Transport Teams in Afghanistan and Iraq; because they were deployed near combat zones, they could rapidly employ life‐saving interventions, likely enhancing battlefield survival rates (Blaz et al., 2013; Byers, 2010; Collins, 2008; Lancaster & Williams, 2010; Peoples et al., 2005). Nurses were especially skilled in preventing deaths from haemorrhage, coagulopathy, acidosis, hypothermia and compartment syndrome, which commonly and concurrently resulted from battlefield traumas (Byers, 2010; Lancaster & Williams, 2010; Peoples et al., 2005). Nurses provided training for local medical services (Lancaster & Williams, 2010; Lewis et al., 2016), such as teaching chest tube insertion and aseptic dressing change procedures to the Afghan National Army and Police; data about local hospital outcomes indicated that this education led to a 45% reduction in their morbidity rates (Lancaster & Williams, 2010). According to Lewis et al. (2016), eight nurse practitioners fulfilled vital roles in the Troop Medical Clinic at Camp Victory Kuwait where they treated an estimated 1956 service members within 1 month for a variety of illnesses and injuries. Nurses were also skilled in managing patients who were suffering from sleep deprivation, depression and acute anxiety (Moore, 2005). According to Peterson et al. (2008), nurses' skills for assessing and managing psychiatric conditions were particularly important during Operation Iraqi Freedom because large numbers of soldiers sustained brain and mutilating injuries caused by improvised explosive devices. Ravella (1995) explained that psychiatric nursing skills were also important in Vietnam because there were soldiers suffering from substance use disorders.

#### 3.3.2 Professional burnout and disillusionment

American military nurses (who were in Afghanistan, Iraq and Vietnam) experienced challenges when they were attempting to reintegrate into their workplaces in the U.S.; the symptoms they reported were consistent with burnout (Doherty & Scannell‐Desch, 2015). “I had had enough,” said an American nurse who worked in the 12th Evacuation Hospital in Củ Chi. A nurse from the 18th Surgical Hospital explained “when I got back [from Vietnam], I was on such overload that I didn't want to have to make another decision the rest of my life. I was tapped out; it was very difficult to deal with the everyday life of home” (DiFilippo, 2017).

For an American nurse who worked in a field hospital in Vietnam “chaos felt normal,” and he struggled with reintegrating into the nursing workforce where there was a much slower pace in the workplace activities (Billie, 1993). Some nurses found it difficult to care for patients who did not have severe injuries because their complaints seemed trivial compared with their wartime patient experiences (Doherty & Scannell‐Desch, 2015; Elliott, 2015; Norman, 1992). Nurses also found it difficult to get along with coworkers when they returned to nursing jobs in the U.S. because the day‐to‐day problems that arose were relatively insignificant, leading them to experience anger and resentment (Doherty & Scannell‐Desch, 2015).

An study conducted by Berger and Gelkopf (2011) demonstrated that educational interventions may reduce burnout in nurses who work in areas frequently impacted by war. They tested a 12‐week programme that provided nurses with education about stress, trauma and resiliency; participation in this programme was associated with significantly decreased burnout, decreased compassion fatigue and increased professional self‐efficacy. Using a feminist perspective, Norman (1992) analysed the post‐war experiences of female Vietnam veterans and concluded that “anger and bewilderment” arose when the women experienced a “loss of status.” According to Norman, women had demonstrated “how they could adapt to the stress of war,” and they were frustrated about being “forced back” into traditional, subservient roles for women when they returned home.

#### 3.3.3 Restricted practice authority

During war, nurses practiced with the full extent of their knowledge and abilities, but they returned to jobs where they had significantly restricted practice authority. Some nurses “questioned their professional worth” (Norman, 1992). A nurse who worked in Vietnam explained feeling “useless” when she returned to a civilian nursing setting due to the laws preventing nurses from practices that had been routine for them during deployment, such as giving blood transfusions (DiFilippo, 2017)—this experience was consistent with the reflections of other American nurses who had been in Vietnam; they felt “devalued” and “underappreciated” (Scannell‐Desch, 1996) and “disillusioned” (Norman, 1992) when they began working as nurses in stateside hospitals. A nurse from the 24th Evacuation Hospital in Long Bình explained that her wartime experience “spoiled me for nursing because I did so much there that I couldn't do in a military hospital in the states; suddenly you weren't allowed to do anything” (Norman, 1992). Nurses in Iran also undertook an augmented scope of practice during war; due to a shortage of physicians, civilian Iranian nurses performed procedures (such as intubations) that were not normally within their scope of practice (Firouzkouhi et al., 2013).

#### 3.3.4 Isolation

Nurses' post‐war experiences illustrated social isolation (Bille, 1993; Conlon et al., 2019; DiFilippo, 2017; Doherty & Scannell‐Desch, 2015; Elliott, 2015; Livingston & Rankin, 1986; Scannell‐Desch, 1996; Wild, 2003). Sometimes, they felt ostracized (Bille, 1993), and other times, they wanted time alone (Ma et al., 2021; Scannell‐Desch, 1996). Nurses described choosing not to share their war experiences with others, and this theme was especially in prominent among American nurses who were in Vietnam. For example, a female nurse explained how her Vietnam war experience became “a hidden part of [her] history” because women were not viewed by society as veterans (DiFilippo, 2017). A male Vietnam veteran did not share his experiences because, upon returning home, he was met with “insults or nothing;” therefore, his war experiences became a “secret” (Bille, 1993). A nurse from the 12th Evacuation Hospital in Củ Chi explained that she “didn't tell anyone [she] was a veteran, let alone a Vietnam veteran; I just packed all that up, stuffed it in a box and put it virtually away” (DiFilippo, 2017). Livingston and Rankin (1986) believed that nurses returning from Vietnam were expected to be “quiet and brave; [they were] not supposed to speak about” the war.

Scannell‐Desch and Doherty (2013) studied families to understand the experiences of nurses who were parents; these nurses reported anxiety about child‐care arrangements during their deployments and fears that their prolonged absence would adversely affect their families. An American nurse in this study explained that the “separation from my kids was the biggest price I paid in this war.” American nurses also explained how they were “not emotionally ready to face their families” upon returning from Vietnam (Scannell‐Desch, 1996). A nurse found it “difficult to fit back into the fabric of [his] family” when he returned to the U.S. from the Middle East (Elliott, 2015), and another nurse's family “could tell he was a changed person. It was the elephant in the room. [They] pretended like nothing had happened” (Doherty & Scannell‐Desch, 2015). Suddenly away from the comradery they developed during war, veterans may have been longing for the “idealized family” that had been comprised of their wartime colleagues, according to Wild (2003). Australian military nurses reported finding it difficult to communicate their wartime nursing stories to people who did not have the same experiences (Conlon et al., 2019), and others described losing friends who could no longer relate to them (Elliott, 2015).

#### 3.3.5 Post‐traumatic stress

Some of the nurses suffered from post‐traumatic stress, and they ruminated about memories of war (Bille, 1993; Brunk, 1997; Doherty & Scannell‐Desch, 2015; Livingston & Rankin, 1986; Ma et al., 2021; Ravella, 1995; Wild, 2003). There were also reports from nurses about feeling “hyper‐vigilant” and unable to sleep (Doherty & Scannell‐Desch, 2015; Elliott, 2015). According to Brunk (1997), nurses' expectations to be nurturing and caring “in the face of war's devastation and carnage” could compound the stress of wartime nursing. Livingston and Rankin (1986) argued that female veterans may have silently suffered from post‐traumatic stress because the U.S. military has “a long history of denying that women” have roles during war; their feminist analysis of American war involvement concluded that the experiences of female veterans have been “invalided” by the myth that women cannot experience war the way men do—women had “nothing to do with the ‘real’ thing,” they argued, but in reality, they had up‐close knowledge of the “death and destruction” (Livingston & Rankin, 1986).

Some American military nurses were hesitant to seek mental health services because they feared how the associated stigma might affect their career options and promotions (Doherty & Scannell‐Desch, 2015). Several former/retired military nurses described how traumatic flashbacks occurred when they witnessed news stories that were similar to their wartime experiences (Bille, 1993; DiFilippo, 2017; Rosenwald, 2021). For example, they vividly remembered soldiers who had died under their care, and they felt guilt (Bille, 1993; Leon et al., 1990; Livingston & Rankin, 1986). An American nurse who worked in the 46th Combat Support Hospital described thinking about the families of soldiers who died in Mogadishu; she “wanted them to know how hard we tried to save their lives, and how we wish we could change the past” (Yoder & Brunken, 2003). An Iranian nurse described war as an “atmosphere in which nurses would pay spiritual and emotional costs” (Rahimaghaee et al., 2016). A feeling of “numbness” was also reported by nurses when they returned home (Bille, 1993; Elliott, 2015; Scannell‐Desch, 1996). An American nurse described feeling “completely numb and empty, like I was looking out of a mask” (Elliott, 2015). Some of the nurses used alcohol to cope (Bille, 1993; Doherty et al., 2020; Livingston & Rankin, 1986; Scannell‐Desch, 1996; Scannell‐Desch, 2000; Wild, 2003).

To understand the characteristics of post‐traumatic stress symptoms among Israeli nurses, Ben‐Ezra et al. (2013) obtained survey data about the effects of the Gaza War. They compared questionnaire scores obtained during the war to scores determined 6 months later in nurses who were exposed versus were not exposed to war. During the Gaza War, the exposed nurses had significantly higher scores for post‐traumatic stress (Impact of Event Scale), depressive symptoms (Center for Epidemiologic Studies Depression Scale), and psychosomatic symptoms (Psychosomatic Problems Scale) compared with nurses who were not exposed to war. By 6 months, however, only the psychosomatic symptom scores remained significantly higher in the exposed group, indicating that these symptoms (such as headaches, stomach aches and sleeplessness) persisted. Ben‐Ezra et al. (2013) concluded that future longitudinal studies should examine the time‐course of nurses' health issues after war to determine the optimal timing for interventions. In a study of Palestinian nurses affected by the Gaza War, Shamia et al. (2015) found that 64% of the nurses reported being traumatized by events occurring in their communities, such as witnessing tanks demolishing their neighbours' homes, illustrating how nurses who are residents of war zones suffer simultaneously from work‐related and community‐related traumas and may therefore require additional interventions to cope with their experiences.

#### 3.3.6 Growth

Many nurses also reflected on the ways that their work had been rewarding, and overall, their experiences contributed to personal growth (Doherty & Scannell‐Desch, 2015; Elliott, 2015; Finnegan et al., 2016; Lev‐Wiesel et al., 2009; Scannell‐Desch, 1996). American nurses described how they eventually “embraced a new normal” after they returned from Vietnam because they “felt forever changed by war.” Doherty et al. (2020) studied American military nurses who served in the Middle East and found that although the nurses reported challenges re‐integrating into civilian life, they experienced “post‐traumatic growth,” which was defined as a “positive psychological change [resulting from] highly challenging life circumstances.” Nurses in this study reported becoming “more compassionate,” developing “personal strength,” and having “a greater appreciation for life.” These qualitative results were corroborated by these nurses' post‐deployment scores on questionnaires, such as the Core Beliefs Inventory and the Posttraumatic Growth Inventory (Doherty et al., 2020). A qualitative study by Elliott (2015) provided similar results; an American nurse described growing and “looking at life through a new lens,” and deployment was defined as “the ultimate experience” despite “so much terrible stuff that happened.” A British nurse who served in Afghanistan reflected that “having that experience, gaining that extra knowledge and toughing it out through the tour makes people grow” (Finnegan et al., 2016). Nurses who worked in northern Israel during the 2006 war with the Hezbollah demonstrated post‐traumatic growth within 2 months of their war experiences; growth was significantly correlated with *personal resources* (defined as protective characteristics that buffer against stress, such as resiliency) and, to a lesser extent, with *dissociation* (defined as a diminished awareness of one's physical and emotional needs; Lev‐Wiesel et al., 2009).

#### 3.3.7 Pacifism

Personal and professional experiences led some nurses to speak out against wars (Boyle & Bunting, 1998; Crowe & Hardill, 1991; Ford, 2022; Gorman, 2004; Tschudin & Schmitz, 2003). Russian nurses recently published a letter opposing war in Ukraine, recounting the loss of “priceless” human lives (Ford, 2022). In the U.S., nurses wrote about the horrors of war that have been faced by civilian populations (Boyle & Bunting, 1998) and by young soldiers and their families (Gorman, 2004)—these nurses called for the profession to focus on pacificism and peace‐keeping and to organize politically to protest wars and protect human rights (Boyle & Bunting, 1998; Gorman, 2004). Canadian nurses, Crowe and Hardill (1991), argued that “health is political,” and they were critical of the way military customs had influenced the nursing profession with “starched uniforms” and “pins signifying rank” while nurses remained “silent on social and political issues.” Livingston and Rankin (1986) defined war as a component of a “patriarchal social structure” where violence is used to “dominate and control,” and combatants “resolve to win, not to resolve conflicts.” Nurses' stories, they argued, “reveal so much about the nature of war.” They saw nurses being forced into a “paradoxical position” because they were “used for [winning] wars” but subsequently became “marginalized and devalued.” Having nurses “patch up the bodies” perpetuated a myth that war was “not about killing people,” they argued (Livingston & Rankin, 1986). Nurses' ethical concerns about sending the “recovered soldiers back to [the] battle” was also a theme that emerged in interviews conducted by Scannell‐Desch (1996). Tschudin and Schmitz (2003) described an ethical duty for nurses in preventing wars, meaning that they must be well‐informed about international events (specifically the hardships faced by refugees) and recognize the profession's “political responsibility.” Boyle and Bunting (1998) also focused on nurses' roles in war‐prevention; they wanted the profession to become more comfortable talking openly about war and not to “turn away.”

### 3.4 Contextual basis: Temporal and sociocultural variations

According to Rodgers, concepts are *evolutionary*, meaning that their definitions can vary over time. Across all of the post‐WWII conflicts that we studied, we found that nurses consistently responded to human suffering and relied on existing resources to support their wartime involvement (i.e., military units and hospitals). Consistent conceptual attributes were also identified across studies (i.e., sacrifice, resourcefulness, tunnel‐vision, survival mindset and comradery). Similar consequences were identified over time and across locations (i.e., reduced morbidity and mortality; a risk for isolation and post‐traumatic stress; possible growth and pacifism) although the professional disillusionment experienced after war may have been the most prominent in the stories told by American Vietnam War veterans. To understand potential sociocultural variations in the concept, we analysed papers about different types of nurses, nationalities and conflicts (e.g., reports from civilians, military officers, volunteers and nurses who experienced attacks on their own communities). We found that nurse from different backgrounds confirmed our definitions of the conceptual antecedents, attributes and antecedents reported in the present paper.

## 4 DISCUSSION

Rodgers' concept analysis methodology was used to determine the ubiquitous/universal attributes of a concept (nurses in conflicts after WWII)—we analysed data over a vast but pertinent timespan (because pivotal professional changes occurred after WWII), and we studied diverse perspectives about wars, which supported similar conceptual definitions. We concluded that nurses' experiences with wars are defined by *sacrifice*, *resourcefulness*, *tunnel‐vision*, *survival mindset* and *comradery*. When wars occurred, nurses were driven to *respond to human suffering* and their involvement was supported by existing *resources*. Nurses' actions *saved lives*. After war, nurses could experience *burnout/disillusionment*, *isolation*, *post‐traumatic stress*, *growth* and *pacifism*. By defining nurses' war‐related experiences, we have synthesized foundational knowledge to inform future research, interventions and policies.

It is important to acknowledge the limitations of paper—we chose not to analyse every available report about nurses' roles after WWII and to instead select a variety of representative papers. This approach could have induced biases, especially because the professional processes for nurses and military operations differ in different countries. For example, the focus on burnout and post‐traumatic stress in the American military nurses could be associated with the requirement for longer deployments in the U.S. compared with other countries. We also examined literature from the entire post‐WWII era, which carries a risk for an anachronistic interpretation of the older papers (i.e., biased by our modern perspective). Only a few of the articles provided data about the nurses' religious and cultural backgrounds, which limited our ability to identify potential variations in the concept according to nurses' cultures. We only selected English language materials; therefore, our findings may not fully reflect the experiences of nurses around the world. Our electronic search strategy may have missed useful resources that were not archived online, and our design did not permit a quantitative meta‐analysis of data across the selected studies.

Our concept analysis has important implications for developing future research programmes, educational and psychosocial support initiatives, and international policies. It will be important for researchers to examine the resourcefulness, innovations and resiliency nurses have developed during wars—this knowledge could improve global procedures for disaster‐preparedness (including systems for responding more effectively to pandemics and bioterrorism). We also found that many nurses would benefit from support mechanisms to prevent post‐traumatic stress, burnout and attrition from the workforce—the profession can begin to invest in this important goal by learning from the experiences of nurses who have practiced during wars. Future research should examine the contextual basis of nurses' experiences during war, such as understanding the differential realities of nurses who volunteer versus nurses who are suddenly thrust into wars when their communities are attacked; knowledge about this topic can determine the support systems these groups require. Finally, our analysis has shown that nurses can educate policy‐makers about the serious and long‐lasting public health consequences of wars—nurses possess crucial knowledge about the consequences of war on global communities, economies and health.

## AUTHOR CONTRIBUTIONS

All authors have agreed on the final version and meet at least one of the following criteria: AMF and GRM: Made substantial contributions to conception and design, or acquisition of data, or analysis and interpretation of data; Involved in drafting the manuscript or revising it critically for important intellectual content; Given final approval of the version to be published. Each author should have participated sufficiently in the work to take public responsibility for appropriate portions of the content; Agreed to be accountable for all aspects of the work in ensuring that questions related to the accuracy or integrity of any part of the work are appropriately investigated and resolved.

## FUNDING INFORMATION

This research received no specific grant from any funding agency in the public, commercial or not‐for‐profit sectors.

## CONFLICT OF INTEREST

No conflict of interest has been declared by the authors.

### PEER REVIEW

The peer review history for this article is available at https://publons.com/publon/10.1111/jan.15454.

## Data Availability

Data sharing not applicable ‐ no new data generated, or the article describes entirely theoretical research.
